# Optimal endoscopic localization of colorectal neoplasms: a comparison of rural versus urban documentation practices

**DOI:** 10.1186/s12957-023-02987-x

**Published:** 2023-03-29

**Authors:** Charbel El-Kefraoui, Garrett Johnson, Harminder Singh, Ramzi M. Helewa

**Affiliations:** 1 Max Rady College of Medicine, University of Manitoba, Winnipeg, MB Canada; 2 Department of Surgery, Section of General Surgery, University of Manitoba, St. Boniface General Hospital, Z3023-409 Tache Avenue, Winnipeg, MB R2H 2A6 Canada; 3 Clinician Investigator Program, University of Manitoba, Winnipeg, MB Canada; 4 Departments of Internal Medicine and Community Health Sciences, University of Manitoba, Winnipeg, MB Canada; 5 CancerCare Manitoba Research Institute, CancerCare Manitoba, Winnipeg, MB Canada

**Keywords:** Colonoscopy, Colorectal cancer, Endoscopy, Repeat preoperative endoscopy, Tattoo localization, Synoptic report

## Abstract

**Background:**

Colonoscopy is the gold standard for diagnosing colorectal neoplasms. However, colonoscopy is often repeated preoperatively due to non-standard documentation and inconsistent practices by index endoscopists. Repeat endoscopies result in treatment delays and can increase risks of complications. National consensus recommendations were recently developed for optimal endoscopic colorectal lesion localization. We aimed to assess baseline colonoscopy practice differences from the new recommendations with a focus on geographical variability in report quality between urban and rural referral sites.

**Methods:**

We performed a retrospective review of patients who underwent elective surgery for colorectal neoplasms at a single institution in Winnipeg between 2007–2020. We compared endoscopy report quality to the national recommendations with charts stratified by endoscopy location. Our primary outcomes were overall report documentation completeness and use of recommended practices.

**Results:**

One hundred ninety-four patients were included (97 rural, 97 urban). The mean overall compliance with the recommendations for urban endoscopies was marginally better compared to rural endoscopies (50% vs. 48%, *p* = 0.04). Sixty-eight percent of the reports complied with tattoo indications (72% urban; 63% rural, *p* = 0.16). On average, reports included 29% of recommended tattoo information (30% urban; 28% rural, *p* = 0.25) and demonstrated 74% appropriate tattoo technique (70% urban; 81% rural, *p* = 0.10). Twenty-one percent of reports included photographs of lesions in accordance with the national recommendations (28% urban; 13% rural, *p* = 0.01).

**Conclusions:**

Endoscopists frequently omit recommended practices for optimal colorectal lesion localization. Rural reports miss more recommended information compared to urban reports. Future research is needed to facilitate province-wide high-quality endoscopy reporting for patients regardless of endoscopy location.

**Supplementary Information:**

The online version contains supplementary material available at 10.1186/s12957-023-02987-x.

## Background

Colorectal cancer is the second leading cause of cancer death in Canada and the United States [1, 2]. Colonoscopies are considered the standard of care for both diagnosis and localization of these lesions prior to surgery [3]. However, localization errors are common [4]. Furthermore, the index endoscopist is often not the surgeon of record. As a result, surgeons often rely on the original endoscopist’s report. Repeat preoperative colonoscopies are one method used to decrease localization errors [4], but they can delay surgery, carry inherent complication risks (i.e., perforation, bleeding), are expensive, and add stress and discomfort to patients [5–7]. Repeat preoperative endoscopy occurs between 28.6% to 40.5% of patients before elective resection for colorectal neoplasms [5, 8, 9]. Tattoo placement, collection of more information for surgical planning, and attempt at endoscopic therapeutic procedures are common reported indications for repeat endoscopies [5, 10, 11]. Therefore, repeat endoscopy could be potentially reduced through the use of consistent tattooing practices and standard documentation of tumor characteristics and location.

In an attempt to enhance colonoscopy practices, new recommendations for the optimal endoscopic localization and documentation of colorectal lesions were recently developed through a Delphi consensus process of leading gastroenterologists and surgeons from across Canada [12]. The goal of these recommendations was to establish standardized practices to decrease tumor localization errors, and to diminish unnecessary repeat preoperative endoscopies. These recommendations also serve as a benchmark against which current endoscopy practices can be measured for the purposes of quality improvement. Therefore, the overarching goal of this paper was to establish a baseline of the quality of endoscopic reports in Manitoba. Specifically, we aimed to assess the extent to which elements documented in the colonoscopy reports for colorectal surgery patients referred to our center differ from the new national consensus recommendations. We hypothesized that many endoscopy reports would have omitted some recommended elements. Furthermore, we hypothesized that report quality would vary between urban and rural locations.

## Methods

### Design and setting

This was a retrospective comparative study using observational data from a random sample of patients who had undergone elective surgery for colorectal lesions at St. Boniface Hospital (SBH) in Winnipeg, Canada between January 1, 2007, and June 30, 2020. The characteristics of these patients, including the repeat preoperative endoscopy rate, nature of the colorectal operation performed, and tumor location, have been described previously [13]. SBH is a tertiary colorectal referral center and receives endoscopy referrals from providers across the entire province of Manitoba. SBH is the primary colorectal surgery center in Manitoba. Manitoba is unique in that there is only a single large urban center (Winnipeg) for the entire province for a population of over 1.4 million people. Moreover, colonoscopies are often conducted by surgeons in rural Manitoba, in comparison to Winnipeg where surgeons and medical endoscopists equally share endoscopic duties.

### Participants

Patient charts were divided into two groups: those where the index colonoscopy was performed in a rural practice setting and those performed in an urban practice setting. Urban endoscopy reports were those generated from within one of the endoscopy suites in Winnipeg. Rural endoscopy reports were those generated from a hospital outside of Winnipeg. Patients were identified from hospital records after their colorectal surgery visit through a combination of admission diagnosis, procedure, and admitting hospital service. Data were extracted from hospital charts and individual surgeon’s private clinical records. Patients were included if they had undergone elective colorectal surgery for a colorectal cancer or adenoma. Operations included: ileocolic resection, right hemicolectomy, extended right hemicolectomy, transverse colectomy, left hemicolectomy, sigmoid resection, anterior resection, low anterior resection, abdominoperineal resection, total abdominal colectomy, and total proctocolectomy. Patients were excluded if they had multiple synchronous tumors, or if they underwent endoscopic excision or transanal endoscopic surgery as their sole surgical treatment. Emergency or palliative surgery patients were also excluded. Prophylactic surgery solely for a genetic colon cancer predisposition, or inflammatory bowel disease, without a distinct polyp or tumor identified preoperatively were also excluded. Small bowel, appendiceal, and anal cancers were excluded.

### Data sources/variables

Data regarding patient characteristics (e.g., age, sex), index endoscopy (e.g., location of index colonoscopy), and surgical procedure (e.g., surgery performed) were obtained from medical records. The quality of endoscopy reports was evaluated against a checklist derived from the new national Delphi consensus recommendations (Additional file 1) [12]. Measures of endoscopy quality included whether the endoscopy procedure was documented in accordance with the recommendations, and whether photographs and tattoos were used when indicated. Placement or omission of tattoos was deemed appropriate based on criteria shown in the Additional file 1. Tattoo information and technique were also assessed. Tattoo technique scores were based on location of the tattoo, number of quadrants involved, volume injected, penetration of the tattoo, and material used to raise the lesion. Appropriate photography contained the following elements (if applicable): lesion before biopsy, anatomic landmarks, tattoo position, and pre-existing tattoos. Data were collected and uploaded digitally on the Research Electronic Data Capture (REDCap) secure online platform [14, 15].

### Sample size

Based on past research conducted by our group, documentation of tattoo placement during colonoscopy for rural patients referred for colorectal surgery was 30%, compared to 60% in urban patients [8]. To observe this same 30% difference for other recommended practices (e.g., lesion photographs, documented polyp characteristics) with 5% alpha and 80% power would necessitate 42 charts per group. To increase the sensitivity of our analysis our study included 97 charts per group to be powered to detect a ≥ 20% difference in completion per variable. Randomization of charts for review was conducted using a random sequence generated in Microsoft Excel Version 16.48 (Microsoft, Redmond, WA).

### Data analyses

All statistical analyses were conducted using STATA 16.1 (StataCorp, College Station, TX). Patient and surgical characteristics were summarized using descriptive statistics. Categorical variables are presented as n (%) and continuous variables are presented as mean (standard deviation, SD) or median (interquartile range, IQR), as appropriate. Our primary analysis was aimed at comparing elements included in endoscopy reports to those recommended by the new consensus recommendations [12]. The total quality score for endoscopy reports was calculated by summing all national recommendations items present in the report and expressing the result as a percentage score. When items were not applicable, their omission did not impact the total score. Secondary analyses explored differences in quality between endoscopy reports completed in an urban center and those completed in a rural center. Welch’s t-test and Pearson’s Chi-squared test were used to determine differences between patient characteristics in each group for continuous and categorical variables, respectively. Mann Whitney U test was used to compare medians. All statistical tests were 2-sided and statistical significance was set at *p* < 0.05.

## Results

A total of 1,690 patients underwent elective surgical resection for colorectal cancer or adenomatous polyps between January 2007 and June 2020. We selected 194 patients (97 urban setting patients and 97 rural setting patients) from this list at random. The baseline and participant characteristics of the included patients are described in Table 1. The mean age was 67 years. Seventy-eight (40%) of the patients were women. One hundred and twelve lesions (58%) were located in the colon, 74 (38%) were located in the rectum, and 8 (4%) were rectosigmoid lesions. There was a significant difference among lesion location between reports done in an urban setting and those done in a rural setting, such that endoscopies conducted in rural settings described more rectal lesions and less colonic lesions.

**Table 1:** Patient and surgical characteristics

	Urban setting	Rural setting	Overall	p*
Number of patients	97	97	194	
Age	68.3 ± 11.2	67.0 ± 16.2	67.6 ± 13.9	0.52
Patients > 75 years old	25 (26%)	32 (33%)	57 (29%)	0.27
Sex				0.38
Female	42 (43%)	36 (37%)	78 (40%)	
Male	55 (57%)	61 (63%)	116 (60%)	
Lesion location				**0.006**
Colon	67 (69%)	45 (46%)	112 (58%)	
Rectum	27 (28%)	47 (48%)	74 (38%)	
Rectosigmoid	3 (3%)	5 (5%)	8 (4%)	
Body Mass Index (BMI)	27.9 ± 3.9	28.6 ± 4.4	28.3 ± 3.4	0.24

Data are expressed as n (%) or mean ± standard deviation (SD)

*p-value is for pairwise comparison between urban and rural settings. Bold indicates statistically significant, *p* < *0.05*

Endoscopy report data compared to recommended practices are described in Table 2. On average, endoscopy reports complied with 49% (95%CI: 47.9%—50.1%) of the recommended practices. Endoscopies generated in urban settings contained more recommended elements compared to those done in a rural setting (50%; 95%CI: 49.1–50.9% vs. 48%; 95%CI: 47.1–48.9%, respectively; *p* = 0.04). Eighty-five endoscopies reported tattoo placement, and on average described 29 ± 12% of recommended tattoo details (i.e., position, number of quadrants tattooed). Thirty reports (15%) mention placing a tattoo distally, 8 reports (4%) mention placing a tattoo proximally, and 4 reports mention placing a tattoo both proximally and distally (2%). Overall, there was no difference between urban and rural reports for completeness of tattoo documentation (30 ± 12% vs. 28 ± 11%, *p* = 0.25).

**Table 2:** Endoscopy report data compared to recommended practices^a^

	Urban setting	Rural setting	Overall	p*
Compliance with tattoo placement indications	70 (72%)	61 (63%)	131 (68%)	0.16
Tattoo information documented (0–100)	30 ± 12	28 ± 11	29 ± 12	0.25
Tattoo technique (0–100)	70 ± 36	81 ± 32	74 ± 34	0.10
Pre-existing tattoo information included (0–100)	40 ± 0	N/A	40 ± 0	N/A
Photography including all elements of recommendations	27 (28%)	13 (13%)	41 (21%)	**0.01**
Lesion characteristics (0–100)				
All colorectal lesions	30 ± 9	30 ± 8	30 ± 8	1.00
Colon lesions	55 ± 22	57 ± 26	56 ± 24	0.56
Rectum and rectosigmoid lesions	24 ± 16	28 ± 14	26 ± 15	0.07
Total score (0–100)	50 ± 4.3	48 ± 4.6	49 ± 4.4	**0.04**

Data are expressed as n (%) or mean ± standard deviation (SD)

^a^Johnson GGRJ, Vergis A, Singh H, Park J, Warriach A, Helewa R [12] Recommendations for optimal endoscopic colorectal lesion localization: A Delphi consensus of national experts. Dis Colon Rectum. In press

^*^p-value is for pairwise comparison between urban and rural settings. Bold indicates statistically significant, *p* < *0.05*

**Fig. 1:**
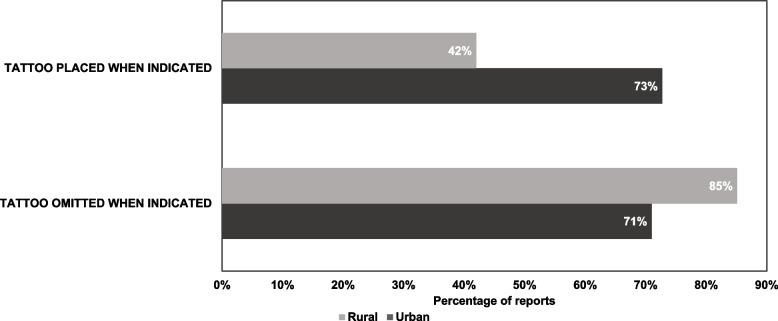
Compliance with tattoo placement indications between urban and rural reports

One hundred and thirty-one (68%) endoscopy reports complied with tattoo placement indication. There was no difference between urban and rural reports in following recommendations for tattoo placement indications (72% vs. 63%, *p* = 0.16). Specifically, 116 endoscopies should have included a tattoo of the lesion. Sixty-nine reports (57%) describe placing a tattoo when indicated by the consensus recommendations. Urban reports were significantly more likely to include a tattoo when indicated (73% vs. 42%, *p* = 0.0008). Furthermore, 78 endoscopies should not have included a tattoo of the lesion. Sixty-two endoscopies (79%) appropriately omitted an endoscopic tattoo when it was not indicated. There was no difference between urban and rural reports (71% vs. 85%, *p* = 0.13) (Fig. 1).

On average, tattoo technique was deemed to be 74 ± 34% appropriate compared to the new recommendations. There was no difference between urban and rural reports (70 ± 36% vs. 81 ± 32%, *p* = 0.10) for tattoo technique. Two endoscopy reports (both done in an urban setting) documented information pertaining to pre-existing tattoos placed at a previous endoscopy. Both reports included 40% of the information presented in the new recommendations for documenting pre-existing tattoos.

Forty-one reports (21%) included photographs of lesions demonstrating all the required information provided in the new recommendations. Urban reports were significantly more likely to include appropriate photographs than rural reports (28% vs. 13%, *p* = 0.01).

On average, reports included 30 ± 8.2% of recommended general lesion feature descriptors. There was no difference between urban and rural reports (30 ± 8.6% vs. 30 ± 7.8%, *p* = 1.00) for all colorectal lesion characteristics. When stratified by colon and rectal lesions, reports included 56 ± 24% of all colon lesion characteristics discussed in new recommendations. There was no difference between urban and rural reports (55 ± 22% vs. 57 ± 26%, *p* = 0.56) in reporting colon lesion characteristics. Reports included 26 ± 15% of rectal and rectosigmoid lesion characteristics discussed in new recommendations. There was no difference between urban and rural reports (24 ± 16% vs. 28 ± 14%, *p* = 0.07) in reporting rectal and rectosigmoid lesion characteristics.

**Fig. 2:**
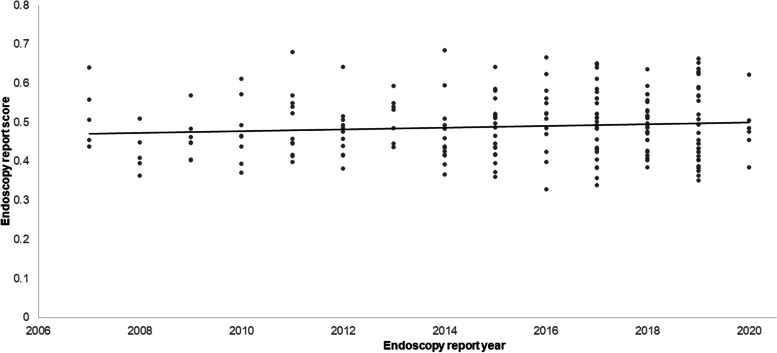
Endoscopy report quality over time

To examine the effect of temporal time trends on report completeness, we performed a post-hoc sensitivity analysis comparing the quality of the reports over time. We found that reports were of consistent quality over time (Fig. 2).

## Discussion

In this study, colonoscopy reports from both urban and rural practice settings showed moderate compliance with the newly derived recommendations. While urban-setting reports were somewhat more compliant, both groups were missing important details pertinent to tattooing information and technique, photography, and general lesion feature description. This deficit in communication is mentioned as an important contributor to repeat endoscopy preoperatively in the literature [5]. Given the differences in quality found between endoscopy reports conducted in urban centers versus rural centers, this might partially help explain differences in rates of repeat preoperative endoscopies that we have previously observed between rural and urban referrals [8].

Several factors may have contributed to our study results. For instance, at our institution in Winnipeg, synoptic reports have been implemented in January 2019. This is done through the EndoVault ® system which includes electronically produced variables of interest, including appending procedure images. This system led to a marked increase in the inclusion of guideline-recommended colonoscopy quality indicators such as photographs [13]. However, endoscopy reports conducted in rural settings in Manitoba remain reliant on narrative reports. Narrative reports are plagued by heterogeneous reporting that differs from one provider to the other [3, 16] Particularly, in narrative reports, the decision to append a photograph to the report is a choice each provider can make. This could explain the significant difference in photography appended to the endoscopy reports in the present study.

With the current trend towards minimally invasive surgeries, appropriate and adequate lesion description and localization is crucial to overcome the inability to rely on tactile stimuli. Yet, inconsistencies in tattooing technique and placement remain common as shown in this study [8, 17]. Additionally, only two endoscopies (both done in an urban setting) documented information pertaining to pre-existing tattoos placed at a previous endoscopy. We suspect that the actual number of pre-existing tattoos present at repeat endoscopies is higher. This may be due to inconsistencies between recommended practices and actual practices [17, 18]. Omitting this information may cause confusion in the operating room, delays in surgery, and resection of erroneous bowel segments.

Overall, when compared to the new recommendations [12], our results show an important deficit between the practices recommended by providers and the practices actually performed. This is likely contributing to the high rate of repeat preoperative endoscopy observed in the literature [5, 8, 9]. Streamlining of endoscopy reports and adherence to a standardized quality checklist in both urban and rural settings might better guide endoscopic reporting, improve standards for quality, and decrease rates of repeat preoperative endoscopy.

Our study needs to be considered in context of its strengths and limitations. To our knowledge, this study is the first of its kind to apply nationwide consensus recommendations for endoscopic lesion localization and it provides baseline information in real world practice for assessing the quality of current endoscopy documentation. Comparing current practices to the new recommendations is an important first step to implement change in provider reporting and improve endoscopic quality. Furthermore, this is a large retrospective observational study which would not be affected by Hawthorne effect of endoscopists perceiving they were under observation. However, there could be confounding variables not considered in the protocol. Moreover, endoscopies conducted in rural settings described more rectal lesions and less colonic lesions. This could be due to referral of more complex rectal lesions to SBH after index endoscopy, while simpler colonic surgeries are performed rurally or at other non-tertiary urban centres. In addition, only a sample of reports randomly selected were analyzed. We do not anticipate any systemic bias from this selection process. Furthermore, despite being adequately powered to detect large differences in quality (i.e., ≥ 20% difference), the study may have lacked sufficient power to detect other smaller, but still significant differences in documentation. Finally, this study was conducted using single-institution data; therefore, our results might not be generalizable to other regions.

## Conclusions

Current endoscopy reports in Manitoba often lack important details and show a slight difference in reporting quality between urban and rural centers. Standardization of reporting according to newly derived recommendations may help improve reporting quality and reduce preventable repeat colonoscopies preoperatively. Given the results of this study, special consideration should be taken to standardize reporting practices across the province, particularly in rural settings, since our results suggest that there is a modestly larger difference between the new recommendations and current practices in rural settings. Future research should also examine the effects of the new recommendations on reporting quality, inter-provider communication and patient outcomes.

## Supplementary Information

**Additional file 1:** Data extraction checklist.

## Data Availability

The datasets used and/or analysed during the current study are available from the corresponding author on reasonable request.
