# Supplementary material for: Optimal endoscopic localization of colorectal neoplasms: a comparison of rural versus urban documentation practices
Source: World J Surg Oncol. 2023 Mar 29;21:115. doi: 10.1186/s12957-023-02987-x (PMC10052793; doi:10.1186/s12957-023-02987-x)
Supplement: Supplementary file 1 — Additional file 1: Data extraction checklist. [file 12957_2023_2987_MOESM1_ESM.docx]

**Online Appendix**

**Table 1. Data extraction checklist derived from the new national Delphi consensus recommendations**

| **Where is the lesion (colon, rectum, rectosigmoid)** |  |
| --- | --- |
| **The following colon lesions should be tattooed:** | |
| Lesion not excised at endoscopy |  |
| Lesion that was partially or incompletely excised at endoscopy |  |
| Lesion to be referred for consideration of surgical resection |  |
| Lesion to be referred for consideration of endoscopic resection (e.g., EMR or ESD) |  |
| Lesion that is difficult to visualize endoscopically |  |
| Lesion that endoscopist wishes to survey in future endoscopic follow-up |  |
| Lesion endoscopist clinically suspects is cancerous |  |
| Lesion after piecemeal polypectomy at endoscopy |  |
| Lesion does not completely lift with submucosal injection |  |
| Lesion ≥2cm |  |
| Lesion ≥3cm |  |
| Circumferential lesion |  |
| Lesion ≥1cm and flat |  |
| Lesion ≥2cm and flat |  |
| Lesion ≥2cm and sessile |  |
| Ulcerated lesion |  |
| Nodular lesion |  |
| Depressed lesion |  |
| Friable lesion |  |
| Firm lesion |  |
| bleeding lesion |  |
| NICE type III lesion |  |
| Large abnormal appearing polyp |  |
| **A tattoo may be safely omitted in the following circumstances** | |
| Small lesions <1cm |  |
| Lesion is invading extra-colonic surrounding structures on imaging |  |
| Pedunculated lesions without other concerning features |  |
| Multiple lesions to be removed in surgical specimen, only the most distal lesion requires a tattoo |  |
| NICE type 1 |  |
| Endoscopist impression is that of a benign lesion |  |
| Elderly or infirm patient in whom future endoscopic surveillance or surgery is not being considered |  |
| **Tattoos of the CECUM should be placed in the following circumstances** | |
| If the endoscopist is not confident of the lesion's location |  |
| If the appendiceal orifice and/or the IC valve are not visualized by the endoscopist with the lesion |  |
| If the appendiceal orifice and/or the IC valve are not photographed with the lesion to prove its location |  |
| **The following RECTAL lesions should be tattooed** | |
| Suspected Rectosigmoid lesions |  |
| Upper rectal lesions |  |
| Suspected rectal neuroendocrine tumors |  |
| **The following information pertaining to tattoos should be documented, if applicable** | |
| Whether a tattoo was placed |  |
| Number of locations (quadrants) injected |  |
| Tattoo placement in relation to lesion |  |
| Tattoo distance from lesion (in cm) |  |
| If tattoo was not placed circumferentially, which side of the bowel was it placed on in relation to lesion |  |
| Whether the endoscopist feels that an accidental transmural injection of ink occurred |  |
| Injection technique (saline bleb technique vs direct injection) |  |
| Type of ink injected |  |
| Volume of Ink injected |  |
| Ink injection angle |  |
| Tattoo placed into the submucosa |  |
| Tattoo used to raise lesion |  |
| Tattoo touches lesion |  |
| Saline injection began prior to inserting needle into submucosa, and advanced until a bleb arises (to decrease risk of going too deep) |  |
| **The following information pertaining to pre-existing (old) tattoos should be documented** | |
| Anatomic location within colon |  |
| Number of spots identified |  |
| Location in relation to lesion |  |
| Tattoo distance from lesion in cm |  |
| If tattoo was not placed circumferentially which side of the bowel was it placed on in relation to lesion |  |
| **Photographs should be used in the following circumstances** | |
| For lesions requiring referral for endoscopic resection |  |
| For lesions requiring surgical resection |  |
| For lesions requiring subsequent endoscopic surveillance |  |
| For all colorectal lesions in which tattoos are placed |  |
| For all colorectal lesions removed at endoscopy |  |
| For all colorectal lesions not removed at endoscopy |  |
| For all colorectal lesions biopsied |  |
| For all colorectal lesions ≥1cm |  |
| For all colorectal lesions except for small benign polyps |  |
| **Photographs should demonstrate the following** | |
| The lesion site post resection |  |
| Important anatomical landmarks in relation to the lesions (if possible) such as IC valve, appendiceal orifice, rectal folds |  |
| Tattoo position in relation to lesion |  |
| Old tattoos that were placed at a prior endoscopy in relation to lesion (if applicable) |  |
| The lesion of interest before biopsy to ensure capture of lesion characteristics (not to obscure by bleeding) |  |
| **For colon lesions only, the following information should be provided** | |
| Colon segment to which the lesion is localized |  |
| Relation to clear anatomical landmarks (e.g., ileocecal valve, appendiceal orifice, hepatic flexure, splenic flexure, etc.) |  |
| Distance from anal verge should be used to document lesions in sigmoid lesions |  |
| Distance from ileocecal valve should be documented for right sided lesions |  |
| Distance from ileocecal valve should be documented for cecal lesions |  |
| **For rectal lesions only, the following information should be provided** | |
| All distance measurements should be recorded on withdrawal |  |
| Distance from anal verge |  |
| Relation to rectal folds |  |
| Distance to top of anorectal ring |  |
| Distance to dentate line |  |
| Whether lesion is palpable |  |
| Laterality |  |
| Anterior vs posterior |  |
| **For all colorectal lesions, the following information should be provided** | |
| Lesion diameter |  |
| Lesion size in relation to reference instrument |  |
| Estimated length of colon involved |  |
| Quadrant(s) involved up to circumferential |  |
| Lesion shape |  |
| Paris classification |  |
| NICE classification |  |
| Ulceration |  |
| Is lesion tethered or mobile |  |
| Friability |  |
| Texture (indurated vs soft) |  |
| Suspected cancer vs benign |  |
| If suspected cancer, for what reason |  |
| Endoscopically resectable |  |
| If endoscopically unresectable, rationale |  |
| If lesion raises with submucosal injection |  |
| If obstructing or near obstructing |  |
| If near obstructing, if able to pass scope beyond |  |
| If near obstructing, diameter of lumen visible |  |
